# Red cell distribution width predicts early kidney injury: A NHANES cross-sectional study

**DOI:** 10.1515/med-2025-1295

**Published:** 2025-10-09

**Authors:** Zixin Chu, Zuojun Liu, Zemin Kuang

**Affiliations:** Healthcare Ward, Beijing Luhe Hospital Affiliated to Capital Medical University, Beijing, 101100, China; Department of Hypertension, Beijing Anzhen Hospital of Capital Medical University, Beijing, 101100, China

**Keywords:** red cell distribution width, early kidney injury, hypertension, NHANES, biomarker

## Abstract

**Objectives:**

To investigate the association between red cell distribution width (RDW) and early kidney injury.

**Methods:**

Data were obtained from the 2003–2004 National Health and Nutrition Examination Survey, including 3,633 adult participants. Early kidney injury was defined according to the 2024 Kidney Disease: Improving Global Outcomes guidelines as eGFR ≥60 mL/min/1.73 m^2^ with urinary albumin-to-creatinine ratio (UACR) 30–300 mg/g, or eGFR 45–60 mL/min/1.73 m^2^ with UACR <30 mg/g. Multivariate logistic regression was used to assess the association between RDW and early kidney injury, adjusting for demographic, socioeconomic, and clinical confounders (age, sex, race, education, poverty index, hypertension, diabetes). Receiver operating characteristic curves were applied to determine the optimal RDW cutoff, and restricted cubic spline (RCS) models were used to explore dose–response relationships.

**Results:**

After adjusting for confounders, there is a positive correlation between RDW and early kidney injury (OR = 1.26, 95% CI: 1.08–1.45, *p* = 0.013). RDW quartile analysis showed that the highest quartile group (>13.1%) had a 1.74-fold risk compared to the lowest group (<12.1%) (95% CI: 1.27–2.40, *p* < 0.001). RCS confirmed a nonlinear dose–response relationship (nonlinear *p* < 0.05). The area under the curve for RDW predicting early kidney injury was 0.86. At the optimal cutoff value of 12.7%, sensitivity was 87.5% and specificity was 71.42%. In the hypertensive population (*n* = 1,190), RDW still significantly predicted early kidney injury (OR = 1.26, 95% CI: 1.10–1.47, *p* = 0.007).

**Conclusion:**

Elevated RDW is significantly associated with the risk of early kidney injury and is robust in the hypertensive population. RDW > 12.7% can serve as an economical and convenient screening threshold, especially suitable for early risk stratification of high-risk groups in resource-limited areas. Future prospective studies are needed to validate its causal mechanism and clinical utility.

## 1 Introduction

Chronic kidney disease (CKD) is a major global public health challenge, with a prevalence of 9.1% in 2017, marking a 29.3% increase since 1990 [1]. Early detection of kidney injury is crucial for preventing progression to end-stage renal disease (ESRD), especially among high-risk populations such as individuals with hypertension [2]. However, conventional renal function biomarkers, such as serum creatinine and estimated glomerular filtration rate (eGFR), often demonstrate limited sensitivity in the early stages of kidney injury, resulting in delayed diagnosis and intervention [3]. Therefore, there is a pressing need for simple, cost-effective, and accessible biomarkers for early screening of kidney damage.

Red cell distribution width (RDW), a routinely reported parameter in complete blood count (CBC) tests, reflects the heterogeneity in red blood cell (RBC) size. Traditionally, RDW has been used in the differential diagnosis of anemia [4]. However, emerging evidence suggests that RDW is associated with various chronic diseases, including cardiovascular disease, malignancies, and chronic obstructive pulmonary disease [5–7]. In nephrology, RDW has been linked to adverse outcomes in patients with CKD and kidney transplant recipients [8,9]. Despite these findings, limited research has focused on the relationship between RDW and early kidney injury, particularly in the general population and high-risk subgroups such as hypertensive patients.

Hypertension is a major risk factor for kidney damage, contributing to approximately 13% of ESRD cases worldwide [10]. Early identification of kidney injury in hypertensive patients is essential to improve clinical outcomes and alleviate the global burden of CKD. Our previous work has shown a significant association between elevated RDW and early renal dysfunction in hypertensive individuals [11]. Building on this, the current study aims to validate the association between RDW and early kidney injury using nationally representative data from the National Health and Nutrition Examination Survey (NHANES) 2003–2004 cycle. Furthermore, we evaluate the predictive utility of RDW in the hypertensive population, offering new insights for clinical early warning and intervention.

This study has several unique strengths. First, it is one of the few large-scale studies to assess the association between RDW and early kidney injury using NHANES data in a diverse population. Second, we propose a clinically applicable RDW cutoff value (>12.7%) to identify individuals at risk, which may serve as a practical screening tool, particularly in settings with limited resources. Finally, our findings highlight RDW as a noninvasive and economical biomarker with potential for early detection of kidney injury, especially in high-risk populations such as those with hypertension.

## 2 Methods

### 2.1 Study population and data source

This study utilized data from the 2003–2004 cycle of the NHANES. The cycle was selected because β2-microglobulin (β2M) was only measured during this period. NHANES is a nationally representative survey conducted by the Centers for Disease Control and Prevention in the United States, aiming to assess the health and nutritional status of the non-institutionalized US civilian population. A stratified, multistage probability sampling design was employed to ensure national representativeness. Data were collected through household interviews, physical examinations, and laboratory tests.

**Figure 1:**
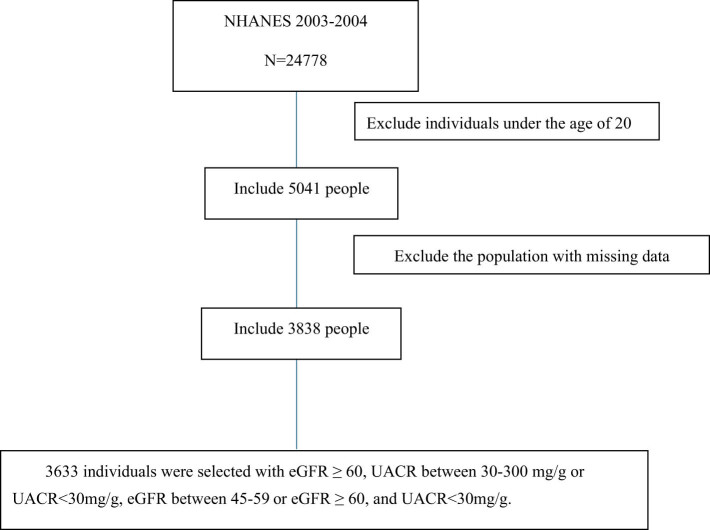
Flowchart of the screening process for selecting qualified participants.

Based on the 2024 Kidney Disease: Improving Global Outcomes guidelines, early kidney injury was defined as eGFR ≥60 mL/min/1.73 m^2^ with urinary albumin-to-creatinine ratio (UACR) between 30 and 300 mg/g or eGFR 45–59 mL/min/1.73 m^2^ with UACR <30 mg/g. The control group was defined as individuals with eGFR ≥60 mL/min/1.73 m^2^ and UACR <30 mg/g. The flowchart for participant screening is shown in Figure 1.

### 2.2 Measurement of RDW

RDW was measured using blood samples collected at mobile examination centers. CBC (including RDW) was performed using a Beckman Coulter MAXM analyzer (Beckman Coulter, Inc., Brea, CA, USA). Individuals who might affect blood sample collection were excluded.

### 2.3 Measurement of kidney function indicators

UACR was calculated as urinary albumin (mg/dL) divided by urinary creatinine (g/dL), using standardized laboratory methods. Serum cystatin C (CysC) andβ2M were measured using the Siemens Dimension Vista 1500 analyzer (Siemens Healthcare Diagnostics, Tarrytown, NY, USA). The eGFR was calculated using the CKD-EPI equation [3].

### 2.4 Covariates

The following covariates were adjusted for in the multivariate analyses: age, sex, race/ethnicity (Mexican American, non-Hispanic Black, non-Hispanic White, other Hispanic, other races), education level (less than 9th grade, 9–11th grade, high school graduate, some college, college graduate or above), and marital status. Poverty–income ratio (PIR), calculated as the ratio of family income to the poverty threshold specific to household size, year, and state. Presence of hypertension and diabetes.

### 2.5 Statistical analysis

All statistical analyses were performed using R software (version 4.4.2). The complex survey design of NHANES was accounted for by applying appropriate sampling weights, stratification, and clustering to ensure nationally representative estimates. Continuous variables were expressed as mean ± standard deviation (SD), while categorical variables were presented as frequencies and percentages. Between-group comparisons were performed using Student’s *T*-test for continuous variables and chi-square tests for categorical variables. The predictive performance of RDW for early kidney injury was evaluated using receiver operating characteristic (ROC) curve analysis, and the area under the curve (AUC) was calculated. To explore the dose–response relationship between RDW and early kidney injury, restricted cubic spline (RCS) models were constructed with knots placed at the 5th, 35th, 65th, and 95th percentiles of RDW distribution. This allowed simultaneous assessment of both linear and nonlinear associations. Statistical significance was set at a two-tailed *p*-value <0.05. Subgroup analyses were conducted, including RDW quartiles and a subgroup of individuals with hypertension. It should be noted that these subgroup analyses were exploratory and were not corrected for multiple comparisons, thus the possibility of type I error (false positives) cannot be ruled out.

## 3 Results

### 3.1 Baseline characteristics of participants

A total of 3,633 participants from the NHANES 2003–2004 cycle met the inclusion criteria and were included in the final analysis. The baseline characteristics are summarized in Table 1. The average age of the participants was 45.48 years (SD = 16.24), and 51.72% were female. The majority were non-Hispanic White (54.09%), followed by Mexican American (20.26%) and other Hispanic groups (18.63%). Significant differences were observed across groups in several variables (*p* < 0.05): participants with early kidney injury were older (mean age = 58.93 years) compared to those without kidney injury (mean = 44.00 years). The early kidney injury group had a lower percentage of higher education attainment. The early kidney injury group had a lower PIR (mean = 2.67) compared to the control group (mean = 3.03). The prevalence of hypertension and diabetes was significantly higher in the early kidney injury group. There were no statistically significant differences in gender, race, or body mass index (BMI) between the two groups.

**Table 1:** Baseline characteristics of participants

		Total	Control group (*n* = 3,143)	Early kidney damage group (*n* = 490)	*p*
Age mean (SD)		45.48 (16.24)	44.00 (15.30)	58.93 (18.29)	<0.0001
Gender *n* (%)					0.349
	Male	1,752 (48.28)	1,527 (87.16)	225 (12.84)	
	Female	1,877 (51.72)	1,615 (86.04)	262 (13.96)	
Race *n* (%)					0.750
	Mexican American	736 (20.26)	644 (87.5)	92 (12.5)	
	Non-Hispanic Black	106 (2.92)	93 (87.74)	13 (12.26)	
	Non-Hispanic White	1,965 (54.09)	1,702 (86.62)	263 (13.38)	
	Other Hispanic	677 (18.63)	577 (85.23)	100 (14.77)	
	Other races	149 (4.10)	127 (85.23)	22 (14.77)	
Education level *n* (%)					<0.0001
	Less than 9th grade	478 (13.17)	381 (79.71)	97 (10.29)	
	9–11th grade	520 (14.33)	440 (84.62)	80 (15.38)	
	High school	910 (25.08)	801 (88.02)	109 (11.98)	
	Some college or AA degree	1,023 (28.19)	899 (87.88)	124 (12.12)	
	College graduate or above	698 (18.23)	621 (88.97)	77 (11.03)	
Family PIR mean (SD)		3.00 (1.60)	3.03 (1.60)	2.67 (1.56)	0.005
BMI mean (SD)		28.22 (6.20)	28.15 (6.19)	28.91 (6.26)	0.178
Diabetes *n* (%)					<0.0001
	Yes	327 (9.13)	214 (65.44)	113 (34.56)	
	No	3,256 (90.87)	2,891 (88.79)	365 (11.21)	
Hypertension *n* (%)					<0.0001
	Yes	1,190 (32.86)	911 (76.55)	279 (23.45)	
	No	2,431 (67.14)	2,220 (91.32)	211 (8.68)	
RDW mean (SD)		12.63 (0.89)	12.58 (0.92)	13.06 (1.34)	<0.0001
UACR mean (SD)		11.50 (22.24)	7.05 (5.19)	52.04 (54.21)	<0.0001
β2M mean (SD)		1.95 (0.54)	1.89 (0.47)	2.46 (0.81)	<0.0001
CysC mean (SD)		0.74 (0.16)	0.72 (0.14)	0.88 (0.24)	<0.0001
eGFR mean (SD)		97.20 (18.56)	99.06 (16.91)	80.26 (23.68)	<0.0001

### 3.2 Association between RDW and early kidney injury

Multivariate logistic regression analyses are presented in Table 2. In the unadjusted model (Model 1), RDW, CysC were all significantly associated with early kidney injury. After adjusting for demographic and socioeconomic factors (Model 2), the association of CysC with early kidney injury was no longer significant. In the fully adjusted model (Model 3), RDW remained significantly associated (OR = 1.32, 95% CI: 1.19–1.46, *p* < 0.001).

**Table 2:** Multivariate logistic regression analysis of RDW and early renal damage prevalence in the general adult population from NHANES 2003–2004

	RDW		CysC		β2M	
	OR (95% CI)	*p*	OR (95% CI)	*p*	OR (95% CI)	*p*
Model 1	1.36 (1.25, 1.50)	<0.001	3.12 (1.02, 9.48)	0.046	1.17 (0.84, 1.638)	0.31
Model 2	1.31 (1.19, 1.46)	<0.001	1.60 (0.47, 5.50)	0.40	1.11 (0.84, 1.48)	0.38
Model 3	1.32 (1.19, 1.46)	<0.001	1.35 (0.35, 5.26)	0.59	1.10 (0.82, 1.48)	0.43

Model 1 is unadjusted. Model 2 is adjusted for covariates including sex, age, race, education level, and poverty–income ratio (PIR). Model 3 further adjusts for hypertension and diabetes in addition to the covariates included in Model 2.

**Figure 2:**
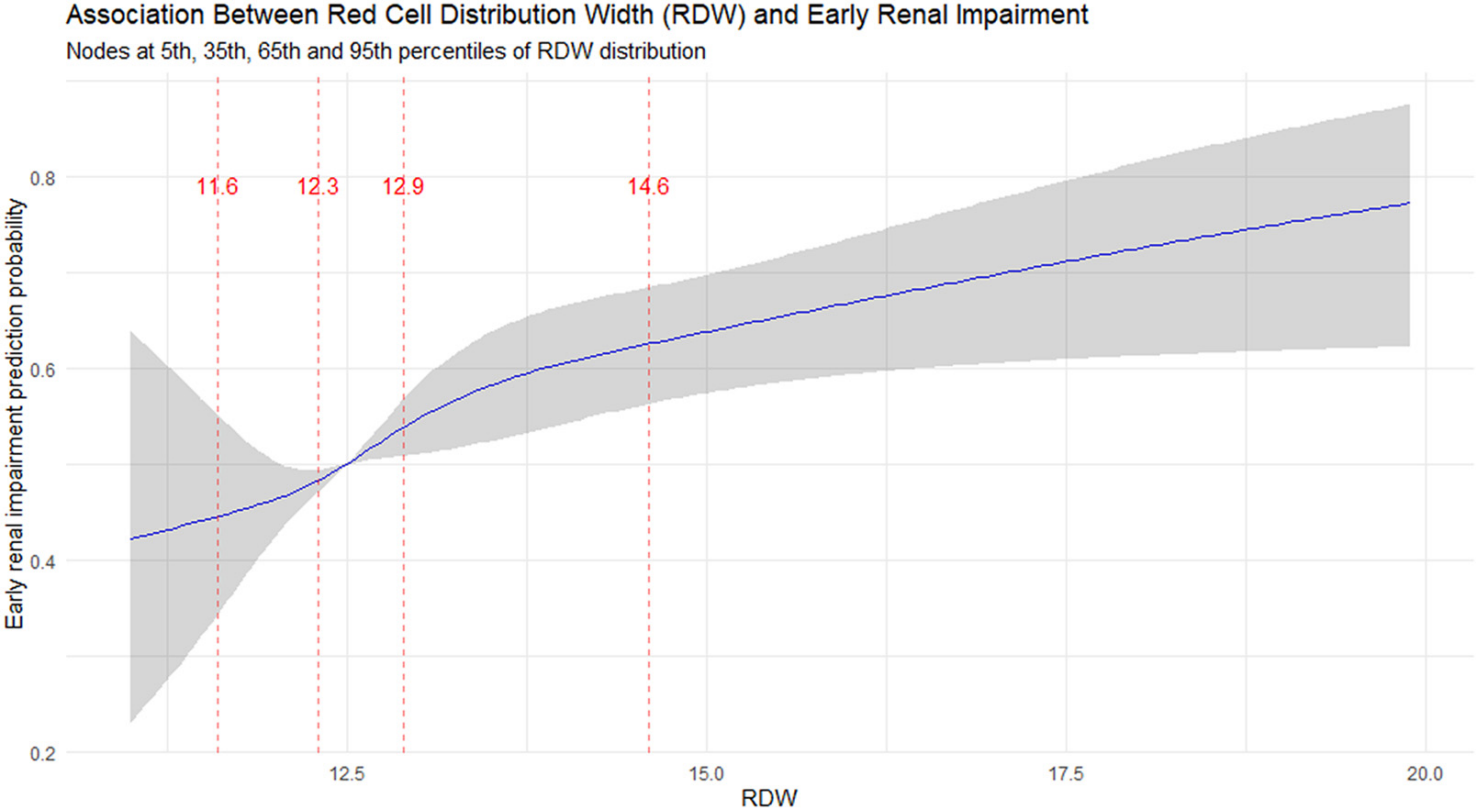
RCS analysis of the relationship between RDW and the predicted probability of early renal damage. The model adjusted for covariates including gender, age, race, education level, poverty index, hypertension, and diabetes, with a nonlinear test *p* < 0.05.

To further examine the dose–response relationship, participants were stratified into quartiles based on RDW (Table 3). Compared with the lowest quartile (<12.1%), those in the highest quartile (>13.1%) had a significantly higher risk of early kidney injury (OR = 1.74, 95% CI: 1.27–2.40). A significant trend was observed across quartiles (*P*_trend_ < 0.001), indicating a progressive increase in risk with rising RDW. RCS analysis with knots at the 5th, 35th, 65th, and 95th percentiles demonstrated a statistically significant nonlinear relationship between RDW and early kidney injury (*p* for nonlinearity <0.05), as illustrated in Figure 2.

**Table 3:** Logistic regression analysis of RDW quartiles and early kidney injury in the general adult population from NHANES 2003–2004

	Quartile 1	Quartile 2 OR (95% CI)	Quartile 3 OR (95% CI)	Quartile 4 OR (95% CI)	*P*_trend_
RDW					
Range	<12.2	12.1–12.5	12.5–13.1	>13.1	
Model 1	1.00	13.07 (0.94, 1.81)	2.32 (1.72, 3.14)	3.20 (2.40, 4.31)	<0.001
Model 2	1.00	0.98 (0.70, 1.38)	1.46 (1.07, 2.02)	1.81 (1.33, 2.50)	<0.001
Model 3	1.00	0.987 (0.70, 1.39)	1.38 (1.00, 1.91)	1.74 (1.27, 2.40)	<0.001

Model 1 is unadjusted. Model 2 is adjusted for covariates including sex, age, race, education level, and poverty–income ratio (PIR). Model 3 further adjusts for hypertension, diabetes, and smoking in addition to the covariates included in Model 2.

### 3.3 Predictive value of RDW

**Figure 3:**
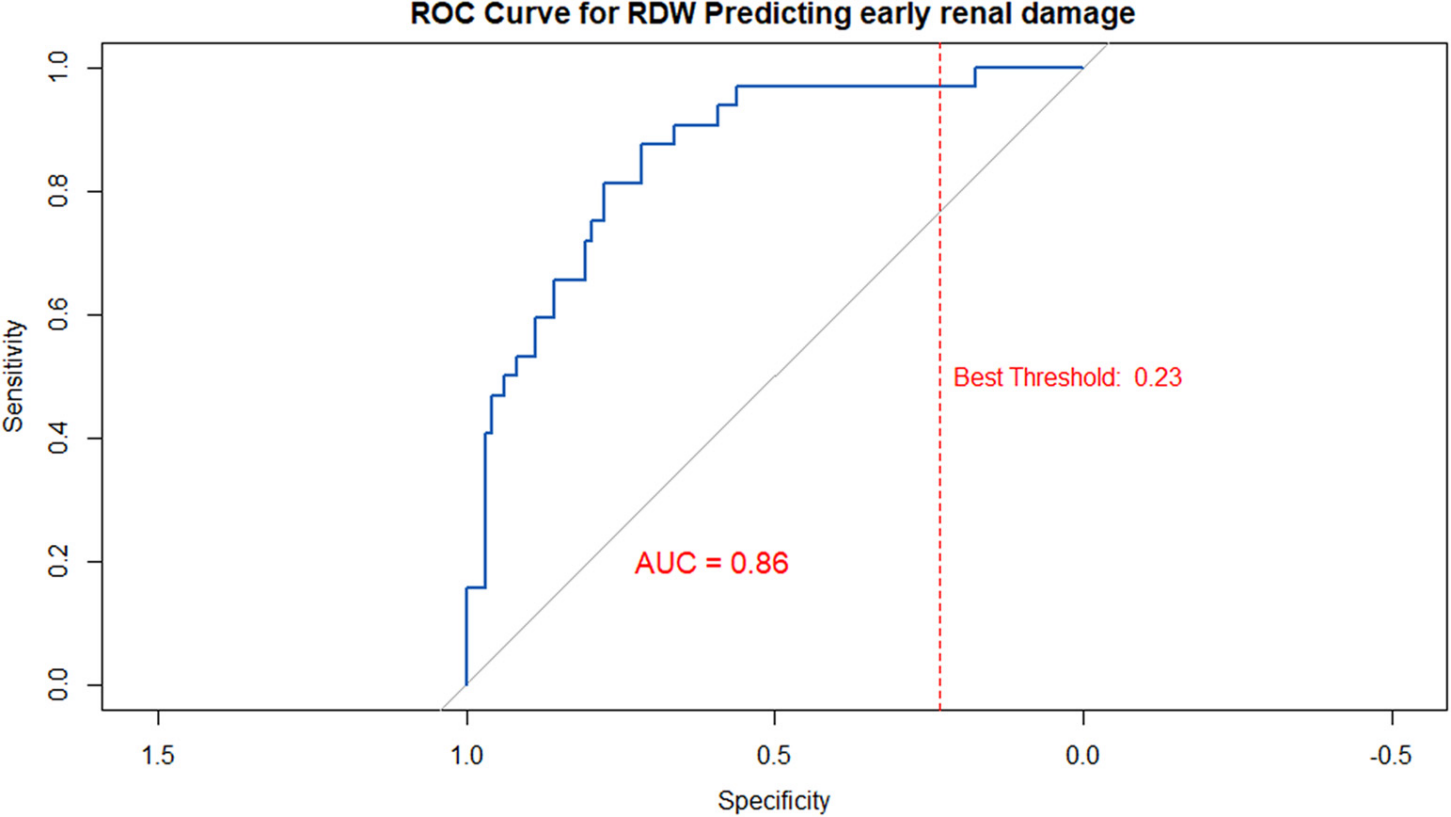
ROC curve for RDW in predicting early renal damage. The model was adjusted for covariates including sex, age, race, education level, PIR, hypertension, and diabetes.

The predictive performance of RDW for early kidney injury was evaluated using ROC curve analysis (Figure 3). The AUC was 0.86, indicating good discriminative ability. The optimal cutoff point was 12.7%, yielding a sensitivity of 87.5% and a specificity of 71.42%.

### 3.4 Subgroup analysis in hypertensive population

Among hypertensive participants (*n* = 1,190), RDW remained a significant predictor of early kidney injury after adjustment for confounders (OR = 1.30, 95% CI: 1.17–1.46, *p* = 0.002). This finding (Table 4) is consistent with previous evidence that RDW is associated with early renal dysfunction in hypertensive individuals [11].

**Table 4:** Multivariate logistic regression analysis of RWD and early renal damage prevalence in hypertensive populations from NHANES 2003–2004

RDW		CysC		β2M	
OR (95% CI)	*p*	OR (95% CI)	*p*	OR (95% CI)	*p*
1.30 (1.17, 1.46)	0.002	1.49 (0.40, 5.58)	0.48	1.09 (0.81, 1.47)	0.46

The model was adjusted for covariates including gender, age, race, education level, poverty–income ratio (PIR), and diabetes.

## 4 Discussion

This study, based on data from the 2003–2004 NHANES cycle, is among the first to demonstrate a significant association between RDW and early kidney injury in a large, diverse adult population. After adjusting for a range of demographic, socioeconomic, and health-related confounders, elevated RDW remained significantly associated with increased risk of early kidney injury (OR = 1.32, 95% CI: 1.19–1.46, *p* < 0.001). These findings underscore the potential of RDW as a simple, cost-effective biomarker for early detection of renal impairment, especially in resource-limited settings.

Through ROC analysis, we identified a clinically applicable RDW threshold of >12.7% for predicting early kidney injury. This threshold demonstrated strong discriminative ability, with an AUC of 0.86, sensitivity of 87.5%, and specificity of 71.4%. However, since the ROC analysis was conducted and tested on the same dataset without cross-validation or external replication, the reported AUC may be overestimated. Therefore, further studies incorporating cross-validation and external validation are essential to confirm the robustness and generalizability of the 12.7% cutoff.

Our findings are consistent with previous research showing that RDW is associated with adverse outcomes in patients with CKD [8] and kidney transplant recipients [9]. However, most existing studies have focused on late-stage CKD or ESRD, with limited data on early kidney injury. This study fills this gap by demonstrating that RDW may also serve as an early indicator of renal impairment, which is critical for timely intervention and improved outcomes [3].

In hypertensive individuals, RDW remained significantly associated with early kidney injury (OR = 1.26; 95% CI: 1.10–1.47; *p* = 0.007), reinforcing previous findings [11] and suggesting that RDW may be a useful marker for risk stratification in this high-risk subgroup. Given the high prevalence of hypertension-related CKD, early detection and intervention in this population are particularly important [10].

Although our primary focus was on RDW, we also evaluated additional kidney-related biomarkers such as CysC and β2M, which were available in the NHANES 2003–2004 cycle. CysC showed higher levels in individuals with early kidney injury; however, neither retained statistical significance in the fully adjusted multivariate models (CysC: OR  =  1.35, *p*  =  0.59; β2M: OR  =  1.10, *p*  =  0.43). This outcome may reflect limited sample size and potential residual confounding. Notably, prior research has indicated that the associations of CysC and β2M with kidney function can be influenced by systemic inflammation, acute infections, and underlying comorbidities [12–14]. Given that RDW, UACR, and eGFR are available across multiple NHANES waves, future analyses should consider integrating data from additional cycles to increase the statistical power and enable robust evaluation of combined biomarker models. Such models incorporating RDW, CysC, and β2M may offer improved predictive accuracy for early kidney injury.

The pathophysiological mechanisms linking elevated RDW and kidney injury are not yet fully understood. Several hypotheses have been proposed: (1) systemic inflammation and oxidative stress: RDW may reflect systemic inflammation and oxidative stress, both of which play pivotal roles in renal injury via pathways involving NF-κB activation and Nrf2 suppression [15]. (2) RBC-derived microparticles (RMPs): RMPs, released during erythrocyte stress or senescence, may contribute to endothelial dysfunction and microvascular damage in the kidneys through pro-inflammatory and pro-coagulant effects [16]. (3) Impaired RBC deformability and hypoxia: elevated RDW may indicate impaired RBC deformability and altered oxygen transport, promoting cortical hypoxia and subsequent glomerular and tubular injury – key drivers of CKD progression. (4) subclinical endothelial dysfunction: RDW has been linked to microvascular dysfunction, potentially mediated by nitric oxide imbalance and reactive oxygen species, which are also implicated in early glomerular injury and proteinuria [17].

We acknowledge several important limitations of this study. Specifically, key confounding variables – such as inflammatory markers (e.g., C-reactive protein), anemia-related indicators (e.g., hemoglobin levels, iron status), and medication use (e.g., ACE inhibitors, ARBs, diuretics) – were either unavailable or incompletely measured in the NHANES 2003–2004 cycle. The absence of these variables may have led to residual confounding, as they could independently influence both RDW levels and kidney function. This limitation underscores the need for caution in interpreting the observed associations and highlights the importance of incorporating a more comprehensive set of covariates in future analyses.

Additionally, RDW is influenced by multiple systemic factors and may serve as a surrogate for underlying inflammation or hematological dysregulation. Future studies should aim to include these additional variables to more precisely assess whether RDW is a causal biomarker of renal injury or merely a reflection of systemic disease processes.

Another limitation is the generalizability of our findings. NHANES data are primarily representative of the US population, with limited representation of Asian and African populations. As such, the utility of RDW as a screening tool in other regions – such as rural China or sub-Saharan Africa – needs further investigation. Variations in genetic background, diet, healthcare, and environmental exposures may influence the predictive value of RDW.

Biomarkers play a critical role in predicting disease occurrence and prognosis in various diseases, such as cancers [18], neurodegenerative diseases [19], and cardiovascular diseases [20]. As a biomarker, the RDW test offers advantages such as low cost, high accessibility, and automation, making it particularly well-suited for use in resource-limited areas. It could be integrated into existing hypertension or diabetes screening programs to identify high-risk individuals who would benefit from further testing, such as UACR or eGFR assessments. Given its high sensitivity (87.5%) and moderate specificity (71.4%), RDW is especially suited as a first-line screening tool in primary care or rural clinics.

Before widespread clinical implementation, two critical validations are needed: external validation of RDW thresholds in diverse populations; prospective studies evaluating whether RDW screening can reduce CKD progression, influence clinical decision-making, or reduce healthcare costs.

## 5 Conclusion

This study found that elevated RDW was significantly associated with an increased risk of early kidney injury, and this association persisted in the hypertensive population. We identified an RDW level >12.7% as a potential clinical threshold for predicting early kidney injury (AUC = 0.86, sensitivity 87.5%, specificity 71.42%). RDW demonstrates strong potential as a screening tool for early kidney injury in resource-limited settings, particularly in primary care facilities. Prospective longitudinal studies in diverse populations are urgently needed to validate the robustness of this RDW threshold, clarify its causal role and underlying biological mechanisms, and assess the real-world impact of RDW-based screening on CKD progression and healthcare resource utilization, ultimately establishing its clinical value as a universal biomarker.
